# Effects of corn straw-based fermented total mixed rations supplemented with exogenous cellulase on growth performance, digestibility, and rumen fermentation in growing beef cattle

**DOI:** 10.5713/ab.24.0246

**Published:** 2024-08-26

**Authors:** Junzhao Xu, Xiaoni Wang, Huaxin Niu

**Affiliations:** 1 College of Animal Science and Technology, Inner Mongolia Minzu University, Tongliao, Inner Mongolia 028000, China

**Keywords:** Chinese Simmental Bull, Corn Straw-based Fermented Total Mixed Ration, Exogenous Cellulase, *In vitro*, *In vivo*

## Abstract

**Objective:**

This study aimed to assess the impact of corn straw-based unfermented and fermented total mixed rations (TMR) supplemented with exogenous cellulase on the *in vitro* fermentation characteristics, growth performance, feeding behavior, apparent digestibility, rumen fermentation and digestive enzyme activities of Chinese Simmental bulls.

**Methods:**

Unfermented (direct spraying of exogenous cellulase onto TMR, TMR) and fermented (exogenous cellulase fermentation for more than 7 d, fermented total mixed rations [FTMR]) TMR were collected, dried, powdered and used as fermentation substrates. The fermentation liquid was ruminal fluid collected from Chinese Simmental bulls. The artificial rumen culture fluid were continuously cultured *in vitro* for 48 h. Based on the diets they were fed, 24 healthy Chinese Simmental bulls (average weight of 495.93±10.89 kg) were randomly divided into two groups, with 12 bulls in each group, which were fed TMR or FTMR. The study lasted 56 d.

**Results:**

In *in vitro* experiments, the neutral detergent fiber (NDF) degradability and total volatile fatty acid, propionate, iso-butyrate, iso-valerate and valerate concentrations were greater in the FTMR group (p<0.05) than in the TMR group. However, the methane production, pH and acetate/propionate (A/P) of the FTMR group tended to be lower (p<0.05) than those of the TMR group. In the *in vivo* experiments, the average daily gain, eating rate, and feed efficiency of the FTMR groups were greater (p<0.05) than those of the TMR group. Similarly, the NDF degradability of the FTMR group was greater (p<0.05) than that of the TMR group. Compared to those in the TMR group, the concentrations of total volatile fatty acids, iso-butyrate, propionate and butyrate were greater in the FTMR group (p<0.05), and the A/P ratio was lower (p<0.05). Similarly, cellulase, xylanase, and β-glucosidase activities were greater (p<0.05) in the FTMR group than in the TMR group.

**Conclusion:**

Corn straw-based FTMR supplemented with exogenous cellulase play a vital role in decreasing the structural carbohydrate content of TMR and ruminal methane production *in vitro*, improving nutrient digestion and absorption, optimizing rumen fermentation, and improving the growth performance of beef cattle.

## INTRODUCTION

The competition between humans and livestock for grain and the effect of imported meat products on the Chinese market have narrowed the profit margins for beef cattle farming, so controlling feed costs is crucial [1]. To overcome this pressure, the Chinese government has actively promoted the “crop straw to beef” program and the “integrated planting and breeding” project to decrease costs and increase efficiency by expanding the whole maize planting area and improving the maize resource utilization rate for feed, thereby decreasing the cost of feed raw materials [2]. The National Bureau of Statistics has defined corn straw as a type of solid organic waste that is difficult to treat and contains cellulose and hemicellulose. Approximately 340 million tons are produced annually in China. With an increase in ruminant breeding, the corn straw utilization rate of feed is also increasing. However, individual fattening households in these corn plantation areas still apply traditional feeding methods and separately feed concentrated feed (broken corn and concentrate) and roughage (low-quality corn straw) [3]. These traditional feeding methods, as well as the complex and dense network structure of cellulose in the cell walls of corn straw, severely restrict and decrease the feed conversion and production efficiency of beef cattle.

Complex cellulase is considered the most promising feed additive at present because it can depolymerize fiber to improve the utilization value of roughage and the digestibility of ruminant feed. However, considering the poor efficacy of direct enzyme administration into the rumen [4], at present, cellulases are utilized as silage additives, alone or combined with other microbiological agents and/or physiochemical treatments; all these strategies facilitate roughage pretreatment, including straw [5]. Several studies have revealed that adding exogenous cellulase can disrupt the surface structure of the cell walls in straw materials, thereby increasing their porosity and specific surface area and allowing the attachment and degradation of rumen microbes; furthermore, this process helps bond breakage between lignin and other fibrous components, thereby decreasing the crystallinity or polymerization degree of cellulose; this promotes cellulose or hemicellulose hydrolysis into more reducing sugars, thereby providing more substrates for lactic acid bacteria fermentation [5–7]. Overall, this process improves feed quality and increases the digestive and absorptive capacity of ruminants. Based on the role of exogenous cellulase in straw feed described above, the use of exogenous cellulase in combination with a fermented total mixed ration (FTMR) is one of the most important methods for improving the feed quality and utilization of corn straw-base total mixed rations (TMR).

FTMR is a kind of feed that combines TMR technology with the principles of silage fermentation. The advantages of FTMR include their small-scale suitability, high nutritional value, high utilization rate, good aerobic stability, complete feed resource utilization, low labor costs, and convenient transport [8]. At present, in Asian countries such as Japan, Korea, and Thailand, studies on FTMR have focused primarily on the fermentation quality and aerobic stability of agricultural byproducts, including cassava pulp [9], broken rice [10], bamboo shoot shell [11], and cottonseed or rapeseed meal [12]. Furthermore, there are studies on the substitution effects of different forage types and the application of additives such as lactic acid bacteria [12] and organic acids [13]. Most of these additives are used to overcome challenges such as nutrient losses owing to an initial absence of lactic acid bacteria, fermentation failures owing to insufficient sugar content in the materials, and quality issues under aerobic conditions [12,13]. Moreover, some studies have been undertaken to evaluate the effects of FTMR produced from agricultural byproducts, forage substitutes, and additives in *in vitro* rumen fermentation experiments [11,13] as well as their application in ruminant production (e.g., sheep [9], cows [14], Hanwoo steers [15], and Thai native beef cattle [10]). However, few studies have comprehensively evaluated the effects of corn straw-based FTMR supplemented with exogenous cellulase on Chinese Simmental bulls from both *in vivo* and *in vitro* perspectives. In this study, we hypothesized that straw-based FTMR supplemented with exogenous cellulase could reduce the structural carbohydrate content of the diet, improve digestibility and the rumen environment, and enhance growth performance and feed efficiency in beef cattle. To this end, we investigated the effects of unfermented TMR (direct spraying of exogenous cellulase onto TMR) and FTMR (exogenous cellulase fermentation for more than 7 d) on the *in vitro* fermentation characteristics, growth performance, apparent nutrient digestibility, rumen fermentation parameters, and digestive enzyme activities of Chinese Simmental bulls.

## MATERIALS AND METHODS

The animal care protocols were approved by the Animal Welfare and Ethics Committee of the College of Animal Science and Technology of Inner Mongolia Minzu University (protocol code: 2020069).

### Diet preparation and sample collection

Corn straw, whole plant corn silage, broken corn, and mixed concentrates were collected from Mengzhiyuan Beef Farm in Tongliao, Inner Mongolia, China. The exogenous enzyme product was obtained from Challenge Co., Ltd., Beijing, China, which was a mixture of cellulase (24,000 U/g), xylanase (40,000 U/g), pectinase (10,000 U/g), and glucanase at a ratio of 2:1:1:1:1. The TMR was formulated to meet the NRC [16] energy and protein requirements of 500 kg for beef cattle. TMR was prepared by mixing all feed ingredients in a TMR mixer (SL-5A; Shandong Xinshengtai Machinery Manufacturing Co., Ltd., Qufu, China). Subsequently, 0.3% exogenous enzymes were added based on dry matter (DM) calculations, and water was added to achieve a moisture content of 50% during processing. The TMR was freshly prepared twice each day. FTMR was prepared by placing the above-prepared TMR in a 200 kg plastic bag, vacuuming it with a domestic vacuum cleaner, and sealing it to form a package. Then, the outer layer of the package was covered with a fiber bag, which was placed in a ventilated warehouse for anaerobic fermentation for more than 7 d (Table 1).

After 7 d of ensiling, the wrapped package was opened. TMR and FTMR samples were collected via the quartering method. A portion of the sample (20 g) was homogenized with distilled water (180 mL) in a blender for 2 min and then filtered through 4 layers of gauze cloth and qualitative filter paper (30 to 50 μm) to obtain the filtrate. A pH meter (pHS-3C; LEICI, Shanghai, China) was used to measure the pH values. The remaining filtrate was centrifuged at 10,000×*g* for 15 min at 4°C, and the organic acid content (lactic acid and acetic acid) was determined by high-performance ion chromatography (ICS-3,000 system; Dionex, Sunnyvale, CA, USA). Organic acids were separated on an AS11 analytical column (250 mm×4 mm) and an AG11 guard column under the following gradient conditions: potassium hydroxide; 0 to 5 min, 0.8 to 1.5 mM; 5 to 10 min, 1.5 to 2.5 mM; and 10 to 15 min, 2.5 mM. The flow rate was 1.0 mL/min. Another portion of the sample (200 g) was dried at 65°C to a constant weight, after which the DM content was determined. The dried samples were ground through a 1 mm screen using a cutting mill (ZM200, Retsch GmbH, Beijing, China) for nutrient analyses and *in vitro* rumen fermentation. The crude protein (CP) content was determined via the Kjeldahl method, and the water-soluble carbohydrate (WSC) content was determined via the phenol-sulfuric acid assay [17]. The method described by AOAC was used to determine the neutral detergent fiber (NDF), acid detergent fiber (ADF), and acid detergent lignin (ADL) contents [17].

### *In vitro* incubation

The TMR and FTMR samples were dried at 65°C and ground using a cutting mill (ZM200; Retsch GmbH, China) to pass through a 1 mm screen. After weighing, 1 g of the dried sample was placed in a preweighed homemade nylon bag (300 mesh). The bag was sealed and put in a 100 mL screw cap fermentation bottle filled with CO_2_. The bottle was then tightly sealed with a single-hole GL45 model plastic cap (the cap was lined with a silicone gasket). The cap was connected to a 200 mL aluminum foil gas collection bag via a silicone tube to collect gas produced during fermentation in real time. The sealed fermentation bottles were then transferred to a 39°C air-bath shaking incubator for preheating. Rumen fluid was collected from three freshly slaughtered Chinese Simmental bulls (approximately 600 kg) from the Mingqing Meat Slaughterhouse (Tongliao, Inner Mongolia, China). The rumen fluid was transferred to preheated, CO_2_-filled thermos flasks and returned to the laboratory. Artificial rumen culture fluid was prepared using Menke’s *in vitro* fermentation method [18]. The rumen fluid was filtered through 4 layers of gauze cloth and mixed with buffer at a ratio of 1:2. The artificial rumen culture fluid was poured into a glass bottle, which was then placed in a 39°C constant-temperature water bath and continuously flushed with CO_2_ to maintain anaerobic conditions. Finally, 80 mL of the artificial rumen culture fluid was added to the preheated fermentation bottles and incubated at 39°C for 48 h in an air-bath shaking incubator at a shaking frequency of 140 rpm. Five replicates were established for each group, with each fermentation bottle representing one replicate, and the experiment was repeated twice. The gas pressure in each bottle was recorded at 3, 6, 9, 12, 24, and 48 h using a syringe to withdraw all the gas from the gas collection bags, which recorded the gas production at each time point from the graduated readings to calculate the final total gas production. At the same time, 10 mL of gas was collected in another gas collection bag for subsequent analysis of methane (CH_4_) gas production. After 48 h of incubation, the bottles were placed on ice to stop fermentation, the pH was measured using the method described above, and the fluid was sampled and preserved at −20°C for the analysis of volatile fatty acid (VFA).

CH_4_ production in the fermentation gas was measured using a high-performance gas chromatograph (TP-2060T; Beijing Beifen Tianpu Instrument Technology Co., Ltd., Beijing, China). The gas was injected using a six-way valve equipped with a thermal conductivity detector. The column used was a TDX-01 column with dimensions of 1 m×3 mm ×2 mm. The column temperature was set to 120°C, the detector temperature to 150°C, and the injection port temperature to 150°C. The carrier gas was argon with a flow rate of 50 mL/min, and the injection volume was 0.1 mL. CH_4_ production (mL) was then calculated based on the actual gas production. After thawing, the samples were centrifuged at 12,000×g for 10 min at 4°C. Then, 1.5 mL of the supernatant was transferred to a 2 mL centrifuge tube, 0.15 mL of 25% metaphosphoric acid was added, and the VFA concentration was measured using a gas chromatograph (model GC-6800; Beijing Beifen Tianpu Instrument Technology Co., Ltd., China). The instrument conditions were as follows: 6 mm×2 m quartz glass packed column, column temperature of 150°C, injection port temperature of 220°C, injection volume of 1 μL, and flame ionization detector temperature of 280°C. The carrier gas was high-purity N_2_ at a 30 mL/min flow rate and a pressure of 200 kPa. The fuel gas was H_2_ at a 30 mL/min flow rate, and the oxidizing gas was air at a 300 mL/min flow rate. After *in vitro* fermentation, the nylon bags from each fermentation bottle were removed and rinsed until clear and odorless. They were then placed in an oven at 105°C and dried to constant weight. The *in vitro* degradability of DM and NDF was calculated as their weight loss after an *in vitro* test.

### Experimental animals

The animal experiment was conducted at the Mengzhiyuan Breeding Cooperative (Tongliao, China). First, 24 Chinese Simmental bulls with an average weight of 495.93±10.89 kg were randomly divided into two groups, with 12 cattle per group, which were fed TMR or FTMR. The daily diet of the cattle was formulated based on the NRC [16]. The TMR was freshly prepared and fed two times each day. FTMR was prepared by wrapping and fermentation for more than 7 d. Table 1 summarizes the nutrient contents and nutrient levels of both diets. All cattle were uniformly managed and housed in individual pens. The TMR and FTMR groups were fed twice daily at 06:30 and 17:30, respectively, with *ad libitum* feeding, and leftover food was controlled at 5% to 10%. The animals had free access to water. The feeding trial was conducted with a 15 d adaptive phase followed by a 56 d experimental period.

### Growth performance and economic benefit

During the formal trial period (1 to 56 d), the amount of feed given and the leftovers were recorded in detail each day, and all the cattle were weighed before feeding on two consecutive mornings at the start of the trial (initial body weight, IBW) and at the end of the trial (final body weight, FBW). The average daily gain (ADG) was calculated using the following equation: (FBW–IBW)/56 d. The average daily feed intake (ADFI) (g/d) for the entire experimental period was calculated from the recorded feed intake of the cattle as follows: total DM intake/number of experimental days. The total DM intake is calculated as the sum of the feed intake over 56 d (feeding amount – remaining amount) multiplied by the DM content of the feed. The feed efficiency (F:G) was calculated using ADG and ADFI as follows: F:G = ADFI/ADG.

Based on the prices of the feed ingredients and the market prices for live cattle at the time, the daily feed costs and the ADG income are calculated, and finally, the daily profits are compared for the two groups. The price of TMR was calculated based on the prices of feed ingredients (DM) and their proportion in the ration for the two groups. The calculation formulas are as follows: daily feed cost (dollar/d/head) = ADFI×TMR price (dollar/DM kg). ADG income (dollar/d/head) = ADG×live cattle price (dollar/kg). Daily profits (dollar/d/head) = ADG income – daily feed cost.

### Feeding behavior

High-definition infrared cameras, barn surveillance cameras, and manual observations on days 54, 55, and 56, respectively, were used to monitor the feed intake and eating behavior of individual beef cattle (5 animals/treatment). The eating behavior of individual beef cattle was described using the following parameters: meal frequency (events/d), meal duration (min/meal), average meal size (kg DM/meal), and eating rate (g DM/min). A meal for individual beef cattle was defined as a visit to the manger followed by an absence from the manger for 300 s or more [19]. The average meal size was calculated by multiplying the amount of feed consumed per meal by the DM content of the feed. The eating rate was calculated by dividing the average meal size by the meal duration.

### Apparent nutrient digestibility

During the test period, feces were collected by rectal sampling at 10:00 and 20:00 on days 54, 55, and 56, with approximately 300 g collected each time. After the last collection, all fecal samples from each bull were mixed equally, and 100 g was placed in a Ziplock bag to which 10 mL of 10% sulfuric acid was added for nitrogen fixation. At the same time, fresh feed samples were collected each day. All collected feces and feed samples were dried in a forced-air oven at 65°C for 72 h and then ground in a mill to pass through a 1 mm screen. The determination of CP, NDF, and ADF in feed and feces was performed according to the methods mentioned above for the determination of feed nutrient composition. The acid-insoluble ash (AIA) content in feed and feces was determined according to the procedure of Van Keulen and Young [20] and used as an indicator for the determination of nutrient digestibility [21].

### Rumen fluid collection, fermentation parameters, and enzyme activity analysis

On 56 d of the experiment, 5 cattle were randomly selected from each treatment group, and approximately 200 mL of ruminal fluid was collected using a stomach tube sampler (GCYQ-1-A; Kelibo Equipment Co., Ltd., Wuhan, China) before the morning feeding. The first 100 mL of the rumen sample was discarded to avoid salivary contamination. A portable pH meter (PHB-4; Laisi Limited Company, Shanghai, China) was used to measure the pH of the rumen. The remaining rumen fluid was transported to the laboratory and stored in a −20°C freezer to determine the VFA content and digestive enzyme activity. The VFA content was determined using the method described in the above *in vitro* test. The activities of rumen digestive enzymes (cellulase, xylanase, and β-glucosidase) were determined by enzyme-linked immunosorbent assay using kits from Jiangsu Enzyme Immunity Industry Co., Ltd. (Jiangsu, China) according to the procedure described by Guo et al [22].

### Statistical analysis

*In vitro* data were statistically analyzed by the MIXED procedure of SPSS 27.0 (version 27.0; IBM, Armonk, NY, USA) with the following model:


$${\text{Y}}_{\text{ij}}=\mu +\tau_{\text{i}}+{ɛ}_{\text{ij}},$$

where Y_ij_ is the response variable, μ is the overall mean, τ_i_ is the experimental diet (i = TMR or FTMR), and ɛ_ij_ is the residual error. *In vivo* data were statistically analyzed by the t-test in SPSS 27.0 (version 27.0; IBM, Armonk, NY, USA). The mean values of the experimental treatments were compared with Tukey’s test. Differences between treatment means were reported as significantly different at p-values <0.05.

## RESULTS

### *In vitro* experiment

After 48 h of *in vitro* incubation, there was no difference in cumulative gas production, DM degradability, or acetate or butyrate concentrations between the two groups. However, NDF degradability and total volatile fatty acid (TVFA), propionate, iso-butyrate, iso-valerate, and valerate concentrations were greater in the FTMR groups (p<0.05) than in the TMR groups. Similarly, the pH and A/P of the TMR group tended to be greater (p<0.05) than those of the FTMR group. Moreover, CH_4_ production after *in vitro* rumen fermentation for 48 h was lower (p<0.05) in the FTMR group than in the TMR group, regardless of whether CH_4_ was expressed based on DM incubation or digestion (Table 2).

### Animal feeding experiment

Notably, the IBW, FBW, and ADFI were similar between the two groups (Table 3). However, the ADG and feed efficiency (F:G) of the FTMR group were greater (p<0.05) than those of the TMR group (Table 3). Furthermore, the daily profits of the FTMR group increased by 9.11% compared to those of the TMR group (Table 3). There was no difference in meal frequency, meal duration or average meal, but the eating rate of the cattle fed the FTMR was greater (p<0.05) than that of the cattle fed the TMR diet (Table 4). The apparent digestibilities of DM, CP, and ADF were similar between TMR and FTMR (Table 5). Nevertheless, the apparent digestibility of NDF was greater (p<0.05) in the FTMR group than in the TMR group (Table 5). Moreover, there was no difference in pH or acetate concentration between the two groups (Table 6). The TVFA, iso-butyrate, propionate, and butyrate concentrations in the FTMR groups were greater (p<0.005) than those in the TMR group. Similarly, the A/P of the FTMR group was lower (p<0.05) than that of the TMR group (Table 6). Moreover, cellulase, xylanase, and β-glucosidase activities were greater (p<0.05) in the FTMR group than in the TMR group (Table 6).

## DISCUSSION

### *In vitro* experiment

The analyses of the effect of TMR and FTMR on *in vitro* digestibility and ruminal fermentation showed that FTMR enhanced NDF digestibility, which may be attributed to the fiber in FTMR containing fewer difficult-to-digest structural carbohydrates. Similarly, Li et al [23] reported that enzymatic digestion can decrease the fiber structure of silage and increase the contact area between rumen microbes and silage, thus improving NDF digestibility. Moreover, the results of this study also showed that CH_4_ production was lower in the FTMR group than in the TMR group, as has been demonstrated *in vitro* by several authors [14,24,25]. In FTMR, the high lactic acid content is converted to propionate by secondary fermentation in the rumen, and the addition of exogenous cellulase can decrease the structural carbohydrates in FTMR, increasing the fermentable substrates and further increasing the propionate content, which consumes hydrogen and can directly inhibit methane production [14, 24,25]. Overall, opposite trends in pH and TVFA concentration were observed during the whole incubation process, which agrees with other reports of *in vitro* experiments [11,13]. The rumen pH can reflect microbial fermentation activity and is closely related to the TVFA content [13,26]. In the present study, the lowest pH value of 6.21 was observed for the FTMR group at the end of incubation, which corresponds to a maximum TVFA concentration of 103.36 mmol/L. Paul et al [24] suggested that reducing pH in the rumen would boost the fermentation process, which would result in increased VFA production. Furthermore, among the VFA components, the propionate content in the FTMR group increased compared to that in the TMR group, and the A/P decreased, which indicates that rumen hydrogen metabolism is more strongly associated with the production of VFA than with the production of methane. This conclusion was preliminarily verified by the reduced methane production observed in this study.

### Animal feeding experiment

The results of the current study showed that the FBW did not differ among dietary treatments, but feeding an FTMR diet to Chinese Simmental bulls increased their ADG and feed efficiency, probably as a result of feeding the lower fiber content of the FTMR. Previous studies have revealed that high-fiber diets have lower digestibility, which reduces feed efficiency and ADG [15]. Using similar diets, Meenongyai et al [27] reported that ADG and feed efficiency improved in Holstein-Zebu cross steers fed an FTMR. Moreover, the ADG results of this study were close to the targets (1.58 to 1.69 kg/d) recommended for Simmental crossbred cattle [28]. We found that the eating rate increased for bulls offered FTMR, which appears to be one explanation for the increased ADG and feed efficiency in the FTMR group and could also be explained by the increase in NDF digestibility in the FTMR group, resulting in increased digestion and absorption of the diet. An increase in the eating rate reflects a decrease in the fiber content of the diet, resulting in enhanced ease of swallowing, increased NDF digestibility, and increased ADG and feed efficiency [19]. Based on the feed and cattle market conditions in Tongliao, Inner Mongolia, during the experimental period, the FTMR group achieved the highest total profit. Furthermore, after deducting feed costs, the daily profit of the FTMR group was 9.11% greater than that of the TMR group.

We observed that the rumen pH of FTMR-fed beef cattle remained stable and unaffected compared with that of TMR-fed cattle, with the pH fluctuating between 6.7 and 7.0. This is an optimal range for the synthesis and activity of composite rumen microorganisms (5.7 to 7.0) [29]. This finding is consistent with that of Supapong et al [29], who reported that FTMR does not modify ruminal pH in Thai beef cattle. In the present study, the diet of the FTMR group improved the TVFA of the bulls compared to the diet of the TMR group, which is consistent with our previous *in vitro* research results, indicating that FTMR supplemented with exogenous cellulase can promote TVFA production [30]. A high-crude fiber diet increases acetate production, whereas a high-nonfibrous carbohydrate diet favors propionate fermentation [31]. Acetate is a precursor for fat biosynthesis in ruminants; propionate is vital for glucose biosynthesis in ruminants, with high propionate levels being beneficial for improving energy utilization in animals [30]. Our results demonstrated that the iso-butyrate, propionate, and butyrate concentrations of bulls fed the FTMR increased compared to those of bulls fed the TMR, with a subsequent decrease in the A/P. The FTMR contains a high amount of lactic acid; hence, as lactic acid is one of the precursors of propionate in rumen, the concentration of propionate is expected to increase [24, 25,32]. In addition, the increase in propionate concentrations might be attributed to the higher non-fiber carbohydrates in the FTMR, which provide more substrates for rumen microbes [24,25,32]. The fed FTMR bulls had higher propionate concentrations, and the acetate concentration remained constant; thus, a lower A/P was expected.

Fiber-decomposing enzymes are needed to degrade the cell wall of forages, and rumen microorganisms can secrete these fiber-decomposing enzymes to hydrolyze the cell wall into sugars and facilitate forage digestion by ruminants [33]. Therefore, the activity of these enzymes may directly reflect the ability of the animal to digest fiber. In the present study, the activities of cellulase, xylanase, and β-glucosidase in the FTMR group were greater than those in the TMR group, which was consistent with the changing trend of NDF digestibility because NDF digestibility mainly depends on the structural carbohydrate content of the diet. Cellulase can disrupt the covalent or noncovalent interactions between cellulose and lignin to generate cellobiose, which is then hydrolyzed by β-glucosidase to release glucose. Xylanase primarily disrupts the glycosidic bonds in hemicellulose xylan to generate xylose and other monosaccharides [34]. The increased activities of these rumen enzymes promote fiber breakdown, thereby improving NDF digestibility. Furthermore, the sugars generated by the hydrolysis of rumen cellulases promoted the metabolism of more propionic acid by microbes, further supporting the conclusion of higher propionic acid levels in the FTMR group. Collectively, these findings suggest that feeding straw-type FTMR containing cellulase can provide more energy to the body, thereby improving the growth performance of beef cattle.

## CONCLUSION

Corn straw-based FTMR supplemented with exogenous cellulase can effectively decrease structural carbohydrate levels in feed, thereby reducing *in vitro* CH_4_ production in the rumen. Furthermore, FTMR can increase *in vitro*/*in vivo* NDF digestibility; increase the concentrations of volatile acids such as propionic acid; and increase cellulase, xylanase, and β-glucosidase activities in the rumen, thereby improving growth performance. In summary, corn straw-based FTMR supplemented with exogenous cellulase provide an effective approach for formulating and utilizing corn straw efficiently in beef cattle diets, demonstrating potential for promotion and application in beef production.

## Figures and Tables

**Table 1:** Ingredients and nutrient levels of the experimental diet (DM basis)

Item	Treatment	

	TMR	FTMR
Ingredient (%)		
Corn straw	25	25
Corn silage	25	25
Broken corn	24	24
Wheat bran	10	10
Soybean meal	6	6
Cottonseed meal	7	7
Urea	0.65	0.65
Limestone	0.75	0.75
NaCl	0.50	0.50
NaHCO_3_	0.35	0.35
CaHPO_4_	0.25	0.25
Premix^1)^	0.50	0.50
Total	100	100
Nutrient levels (%)		
DM	54.39	53.01
CP	12.12	12.04
NDF	40.30	37.96
ADF	23.21	19.60
ADL	2.23	2.05
Hemicellulose^2)^	18.09	17.36
Cellulose^3)^	20.98	17.55
NE_mf_ (MJ/kg)^4)^	6.58	6.52
Fermentation profile (%)		
WSC	6.50	4.70
DM loss	-	1.36
pH	5.25	4.33
Lactic acid	1.60	7.58
Acetic acid	1.02	6.53

DM, dry matter; TMR, total mixed ration; FTMR, fermented total mixed ration; CP, crude protein; NDF, neutral detergent fiber; ADF, acid detergent fiber; ADL, acid detergent lignin; WSC, water-soluble carbohydrate; NE_mf_, comprehensive net energy.

1) Composition of premix. Per kg: copper 650 mg, iron 3,200 mg, manganese 1,500 mg, zinc 2,000 mg, cobalt 10 mg, selenium 10 mg, vitamin A 1,500,000 IU, vitamin D 320,000 IU, vitamin E 3,000 IU.

2) Hemicellulose = NDF–ADF.

3) Cellulose = ADF–ADL.

4) The NE_mf_ was calculated according to the NRC (2000), while the others were measured values.

**Table 2:** Gas production, degradability, and fermentation parameters of the TMR and FTMR groups after 48 h of *in vitro* ruminal incubation

Items	Groups		SEM	p-value

	TMR	FTMR		
Total gas (mL/g DM)	275.67	276.0	3.13	0.917
Degradability (%)				
DM degradability	79.96	81.02	0.90	0.270
NDF degradability	68.88^b^	72.31^a^	0.63	<0.001
CH_4_ production				
CH_4_ (mL/g DM)	40.11^a^	38.37^b^	0.46	0.004
CH_4_ (mL/g DMD)	50.16^a^	47.36^b^	0.34	<0.001
Fermentation characteristics				
pH	6.31	6.21	0.03	<0.001
TVFA (mmol/L)	101.22^b^	103.36^a^	0.45	<0.001
Acetate (mol/100 mol)	63.16	62.42	0.39	0.089
Propionate (mol/100 mol)	19.3^b^	20.46^a^	0.19	<0.001
Iso-butyrate (mol/100 mol)	1.51^b^	1.75^a^	0.04	<0.001
Butyrate (mol/100 mol)	12.30	12.91	0.31	0.084
Iso-valerate (mol/100 mol)	3.15^b^	3.68^a^	0.07	<0.001
Valerate (mol/100 mol)	1.78^b^	2.13^a^	0.04	<0.001
A/P	3.27^a^	3.05^b^	0.04	<0.001

TMR, total mixed ration; FTMR, fermented total mixed ration; SEM, standard error of the mean; DM, dry matter; NDF, neutral detergent fiber; CH_4_, methane; TVFA, total volatile fatty acid; A/P, acetate/propionate.

a,b In the same row, values with different superscripts differ significantly (p<0.05).

**Table 3:** Effects of TMR and FTMR on the growth performance and economic benefit of beef cattle^1)^

Items	Groups		SEM	p-value

	TMR	FTMR		
IBW (kg)	497.86	498.14	5.66	0.961
FBW (kg)	587.71	599.10	7.65	0.162
ADG (kg)	1.68^b^	1.80^a^	0.03	0.002
ADFI (kg)	11.75	11.98	0.15	0.162
F:G	7.03^a^	6.66^b^	0.01	<0.001
Feed input (dollar/DM kg)	0.31	0.32	-	-
Daily feed cost (dollar/d/head)	3.64	3.83	-	-
Income of daily weight gain (dollar/d/head)	7.93	8.50	-	-
Daily profits (dollar/d/head)	4.28	4.67	-	-

TMR, total mixed ration; FTMR, fermented total mixed ration; SEM, standard error of the mean; IBW, initial body weight; FBW, final body weight; ADG, average daily gain; ADFI, average daily feed intake; F:G, feed efficiency = ADFI/ADG.

1) From June to September 2021, the prices for corn straw and whole corn plant silage were 0.11 and 0.08 dollar/DM kg, respectively. The prices for other materials were as follows: broken corn, wheat bran, soybean meal, cottonseed meal, urea, limestone, NaCl, NaHCO_3_, CaHPO_4_ and premix were 0.31, 0.36, 0.54, 0.44, 0.33, 0.04, 0.11, 0.31, 0.33, and 1.67 dollar/DM kg, respectively. The price of silage bagging is 0.01 dollar/DM kg. The market price of live cattle in Tongliao, Inner Mongolia, in September 2021 was 4.72 dollar/kg.

a,b In the same row, values with different superscripts differ significantly (p<0.05).

**Table 4:** Effects of TMR and FTMR on the feeding behavior of beef cattle

Items	Groups		SEM	p-value

	TMR	FTMR		
Meal frequency (events/d)	8.89	8.11	0.37	0.051
Meal duration (min/meal)	19.78	18.67	0.55	0.059
Average meal size (kg DM/meal)	1.32	1.35	0.03	0.248
Eating rate (g DM/min)	66.85^b^	72.07^a^	2.33	0.040

TMR, total mixed ration; FTMR, fermented total mixed ration; SEM, standard error of the mean.

a,b In the same row, values with different superscripts differ significantly (p<0.05).

**Table 5:** Effects of TMR and FTMR on apparent digestibility in beef cattle

Items	Groups		SEM	p-value

	TMR	FTMR		
DM (%)	70.25	71.88	1.25	0.262
CP (%)	67.43	68.37	0.93	0.372
NDF (%)	51.88^b^	58.90^a^	2.66	0.040
ADF (%)	45.44	48.33	2.95	0.383

TMR, total mixed ration; FTMR, fermented total mixed ration; SEM, standard error of the mean; DM, dry matter; CP, crude protein; NDF, neutral detergent fiber; ADF, acid detergent fiber.

a,b In the same row, values with different superscripts differ significantly (p<0.05).

**Table 6:** Effects of TMR and FTMR on rumen fermentation characteristics and enzyme activities in beef cattle

Items	Groups		SEM	p-value

	TMR	FTMR		
Fermentation characteristics				
pH	6.97	6.87	0.47	0.101
TVFA (mmol/L)	82.27^b^	91.19^a^	1.50	0.004
Acetate (mol/100 mol)	50.69	51.53	0.51	0.173
Propionate (mol/100 mol)	17.04^b^	19.90^a^	0.37	0.002
Iso-butyrate (mol/100 mol)	1.74^b^	2.52^a^	0.11	0.002
Butyrate (mol/100 mol)	10.36^b^	14.21^a^	1.27	0.038
Valerate (mol/100 mol)	2.43	3.02	0.37	0.183
A/P	2.97^a^	2.59^b^	0.07	0.007
Enzyme activities				
Cellulase (IU/L)	17.81^b^	19.70^a^	0.33	0.004
Xylanase (U/L)	91.41^b^	119.59^a^	0.88	<0.001
β-glucosidase (U/L)	187.59^b^	240.69^a^	4.15	<0.001

TMR, total mixed ration; FTMR, fermented total mixed ration; SEM, standard error of the mean; TVFA, total volatile fatty acid; A/P, acetate/propionate.

a,b In the same row, values with different superscripts differ significantly (p<0.05).

## References

[1] Li XZ, Yan CG, Zan LS (2018). Current situation and future prospects for beef production in China — a review. Asian-Australas J Anim Sci.

[2] Li Y, Shi Y, Deng X, Sun Z, Accatino F (2024). Increasing food and feed self-sufficiency and avoiding manure N surplus in eastern regions of China through a spatial crop-livestock optimisation model. Agric Syst.

[3] Wang C, Zhang C, Yan T (2020). Increasing roughage quality by using alfalfa hay as a substitute for concentrate mitigates CH4 emissions and urinary N and ammonia excretion from dry ewes. J Anim Physiol Anim Nutr.

[4] Elwakeel EA, Titgemeyer EC, Johnson BJ, Armendariz CK, Shirley JE (2007). Fibrolytic enzymes to increase the nutritive value of dairy feedstuffs. J Dairy Sci.

[5] Jabri J, Abid K, Yaich H, Malek A, Rekhis J, Kamoun M (2022). Evaluation of the efficacy of varying xylanase to cellulase ratio on ruminal fermentation of untreated and alkali treated oat straw. Res Sq.

[6] Nie D, Yao L, Xu X, Zhang Z, Li Y (2021). Promoting corn stover degradation via sequential processing of steam explosion and cellulase/lactic acid bacteria-assisted ensilage. Bioresour Technol.

[7] Zhang G, Fang X, Feng G, Li Y, Zhang Y (2020). Silage fermentation, bacterial community, and aerobic stability of total mixed ration containing wet corn gluten feed and corn stover prepared with different additives. Animals.

[8] Bueno AVI, Lazzari G, Jobim CC, Daniel JLP (2020). Ensiling total mixed ration for ruminants: a review. Agronomy.

[9] Khejornsart P, Meenongyai W, Juntanam T (2022). Cassava pulp added to fermented total mixed rations increased tropical sheep’s nutrient utilization, rumen ecology, and microbial protein synthesis. J Adv Vet Anim Res.

[10] Kotupan S, Sommart K (2021). Broken rice in a fermented total mixed ration improves carcass and marbling quality in fattened beef cattle. Anim Biosci.

[11] Zhao J, Wang S, Dong Z, Chen L, Shao T (2021). Partial substitution of whole-crop corn with bamboo shoot shell improves aerobic stability of total mixed ration silage without affecting in vitro digestibility. J Anim Physiol Anim Nutr.

[12] Yusuf HA, Piao M, Ma T, Huo R, Tu Y (2022). Effect of lactic acid bacteria and yeast supplementation on anti-nutritional factors and chemical composition of fermented total mixed ration containing cottonseed meal or rapeseed meal. Anim Biosci.

[13] Chen L, Yuan X, Li J (2019). Effects of applying lactic acid bacteria and propionic acid on fermentation quality, aerobic stability and in vitro gas production of forage-based total mixed ration silage in Tibet. Anim Prod Sci.

[14] Arangsri M, Pattarajinda V, Duangjinda M, Mungkalasiri W, Angthing JK (2019). Impact of fermented total mixed rations on intake, VFA and methane production of dairy heifers. Indian J Anim Res.

[15] Kim T, Mayakrishnan V, Lim D, Yeon JH, Baek KS (2018). Effect of fermented total mixed rations on the growth performance, carcass and meat quality characteristics of Hanwoo steers. Anim Sci J.

[16] National Research Council (2001). Nutrient requirements of dairy cattle.

[17] AOAC (2019). Association of Official Analytical Chemists Official methods of analysis.

[18] Menke KH, Raab L, Salewski A, Steingass H, Fritz D, Schneider W (1979). The estimation of the digestibility and metabolizable energy content of ruminant feedingstuffs from the gas production when they are incubated with rumen liquor in vitro. J Agric Sci.

[19] Koenig KM, Chibisa GE, Penner GB, Beauchemin KA (2020). Optimum roughage proportion in barley-based feedlot cattle diets: growth performance, feeding behavior, and carcass traits. J Anim Sci.

[20] Van Keulen J, Young BA (1977). Evaluation of acid-insoluble ash as a natural marker in ruminant digestibility studies. J Anim Sci.

[21] Chumpawadee S, Leetongdee S (2020). Effect of level of cassava pulp in fermented total mixed ration on feed intake, nutrient digestibility, ruminal fermentation and chewing behavior in goats. Songklanakarin J Sci Technol.

[22] Guo G, Shen C, Liu Q (2020). The effect of lactic acid bacteria inoculums on in vitro rumen fermentation, methane production, ruminal cellulolytic bacteria populations and cellulase activities of corn stover silage. J Integr Agric.

[23] Li J, Yuan X, Dong Z, Mugabe W, Shao T (2018). The effects of fibrolytic enzymes, cellulolytic fungi and bacteria on the fermentation characteristics, structural carbohydrates degradation, and enzymatic conversion yields of pennisetum sinese silage. Bioresour Technol.

[24] Paul OB, Urmi SS, Biswas MAA (2023). Effect of TMR and fermented TMR on ruminal in vitro digestion and gas production. Adv Anim Vet Sci.

[25] Wang C, Uyeno Y, Jayanegara A, Kondo M, Ban-Tokuda T, Matsui H (2016). Changes in in vitro rumen fermentation characteristics of different compositions of total mixed rations (TMR) and the ensiled TMRs. Adv Anim Vet Sci.

[26] Li Y, Lv J, Wang J (2021). Changes in carbohydrate composition in fermented total mixed ration and its effects on in vitro methane production and microbiome. Front Microbiol.

[27] Meenongyai W, Pattarajinda V, Stelzleni AM, Sethakul J, Duangjinda M (2017). Effects of forage ensiling and ration fermentation on total mixed ration PH, ruminal fermentation and performance of growing Holstein-Zebu cross steers. Anim Sci J.

[28] Wang C, Wang Z, Hu R (2021). Effects of different types of white distiller’s grains on growth performance, nutrient apparent digestibility, serum biochemical indexes and rumen fermentation parameters of simmental crossbred cattle. Chin J Anim Nutr.

[29] Supapong C, Cherdthong A, Wanapat M, Chanjula P, Uriyapongson S (2019). Effects of sulfur levels in fermented total mixed ration containing fresh cassava root on feed utilization, rumen characteristics, microbial protein synthesis, and blood metabolites in Thai native beef cattle. Animals.

[30] Yusuf HA, Rehemujiang H, Ma T, Piao M, Huo R, Tu Y (2022). Fermented total mixed ration with cottonseed meal or rapeseed meal improved growth performance and meat quality of hu lamb compared to total mixed ration with soybean meal. Fermentation.

[31] Lechartier C, Peyraud JL (2010). The effects of forage proportion and rapidly degradable dry matter from concentrate on ruminal digestion in dairy cows fed corn silage–based diets with fixed neutral detergent fiber and starch contents. J Dairy Sci.

[32] Cao Y, Takahashi T, Horiguchi K, Yoshida N, Cai Y (2010). Methane emissions from sheep fed fermented or non-fermented total mixed ration containing whole-crop rice and rice bran. Anim Feed Sci Technol.

[33] Mizrahi I, Wallace RJ, Moraïs S (2021). The rumen microbiome: balancing food security and environmental impacts. Nat Rev Microbiol.

[34] Zayed MS, Szumacher-Strabel M, El-Fattah DA (2020). Evaluation of cellulolytic exogenous enzyme-containing microbial inoculants as feed additives for ruminant rations composed of low-quality roughage. J Agric Sci.

